# The Leading Factors of Obesity and Severe Obesity in Korean Adults during the COVID-19 Pandemic

**DOI:** 10.3390/ijerph191912214

**Published:** 2022-09-26

**Authors:** Myung-Nam Lee, Young-Soon Choi, Sang-Dol Kim

**Affiliations:** Department of Nursing, College of Health Science, Kangwon National University, 346 Hwangjo-gil, Dogye-eup, Samcheok-si 25949, Kangwon-do, Korea

**Keywords:** COVID-19 pandemic, obesity, severe obesity, sitting time, walking time, energy intake

## Abstract

(1) Background: During the coronavirus disease 2019 (COVID-19) pandemic, the prevalence of obesity or severe obesity has increased worldwide to the point that it has even been referred to as a new disease. However, the impacts of the pandemic on obesity or severe obesity remain unclear, thus requiring a thorough examination of the leading factors of obesity and severe obesity during this time. (2) Methods: The required dataset for this study was extracted from the eighth (2019–2020) Korea National Health and Nutrition Examination Survey (KNHNES). The survey’s data for 2019 and 2020 were analyzed to confirm the leading factors of obesity and severe obesity before and after the outbreak of COVID-19. The samples were weighted, and the data were analyzed using multiple logistic regression. (3) Results: In 2020, the prevalence of obesity and severe obesity in the Korean adult population aged 19 and over, compared with the normal weight group, showed significant increases of 2.5% and 1.4%, respectively, compared with those rates in 2019 (*p* < 0.05). The main variables affecting the obesity prevalence in Korean adults aged 19 and over in 2020 were gender, age, sitting time per day, and walking time per day, and the factors affecting severe obesity were gender and age. Meanwhile, the daily energy intake variable had no impact on the prevalence of obesity and severe obesity. (4) Conclusions: These findings will serve as a basis to help the present management directions and treatment approaches for individuals with obesity or severe obesity in the post-COVID-19 era.

## 1. Introduction

Humans were placed in a pandemic situation caused by coronavirus disease 2019 (COVID-19) at the end of 2019. This resulted in sudden and drastic changes in their daily lives and working patterns, including self-quarantine, social distancing, increased sedentary behaviors, decreased physical activity, dietary changes, and emotional disturbances [1,2,3]. A remarkable aspect of the COVID-19 outbreak has been the rapid weight gain, obesity, and severe obesity in individuals or populations [4,5,6]. A prior study indicated that the COVID-19 pandemic has been identified as a contributing factor that can accelerate weight gain in humans [7]. Furthermore, another study observed that weight gain can negatively affect human health and lead to a predisposition to the stigma associated with weight, such as obesity and high obesity [6]. Other previous studies stressed that obesity and severe obesity lead to immune vulnerabilities and cause respiratory diseases, which have been described as new diseases as a result of the COVID-19 pandemic [4,8,9,10,11]. Based on the discussion of previous studies, weight gain and the COVID-19 pandemic are considered to be involved in a vicious cycle. In other words, the COVID-19 pandemic made it necessary for people to self-quarantine, resulting in weight gain and thus repeating the cycle of obesity and severe obesity [12,13]. In the post-COVID-19 era, obesity and severe obesity have, therefore, become increasingly challenging issues [11,14]. The general opinion is that weight gain, a leading cause of obesity and severe obesity, should be suppressed in order to break this chain.

Accordingly, previous studies have primarily focused on the population of each country to obtain representative data, and they have explored and reported the leading factors contributing to weight gain during the COVID-19 pandemic [1,2,15,16,17,18]. Several previous studies have also examined variables such as gender, age, dietary habits, and physical activities as the determinants of weight gain caused by the outbreak of COVID-19 [2,15,19,20]. According to previous studies, sharing representative data from around the world may help alleviate and prevent weight gain in the event of similar global events or diseases.

However, the focus was primarily on the topic of weight gain, and references to obesity and severe obesity problems that required urgent attention were passively discussed. Thus, it is suggested that the factors affecting obesity and severe obesity as a result of self-quarantine due to the COVID-19 pandemic should also be specifically identified as early as possible. Therefore, this study aims to highlight the leading factors of obesity and severe obesity in Korean adults aged 19 and over by comparing the data before and after the outbreak of COVID-19.

## 2. Materials and Methods

### 2.1. Study Design and Participants

This was a secondary data analysis, based on raw data with secured representation through a two-stage stratified sampling method. The raw dataset was obtained from the 8th Korea National Health and Nutrition Examination Survey (KNHANES) (2019–2020) by the Korea Disease Control and Prevention Agency (KDCA) [21]. 

For the KNHANES, the target group was the citizens aged one or older living in Korea, and sample extraction was performed using a two-stage stratified cluster sample extraction method using survey ports and households as the first and second extraction units, respectively. The survey was stratified based on the first standard for stratification (city, housing type), the second standard for stratification (residential area ratio), and the intrinsic standard for stratification (householder education ratio) to extract 576 survey districts over a period of three years. In the sample survey, 25 sample households were selected using the systematic extraction method among appropriate households, excluding facilities such as nursing homes, military and prison facilities, and foreign households. The survey included all household members aged one or older within the sample households. Despite the COVID-19 pandemic, for the eighth 2nd-year survey (2020), 180 health surveys (93.8% completion rate) and 166 nutrition surveys (86.5% completion rate) were completed out of the 192 survey districts nationwide.

The total number of respondents was 12,218, including 6296 in 2019 and 5922 in 2020. In this study, the sample was restricted to those individuals aged 19 years and older, with the exception of pregnant women.

### 2.2. Surveying the Amount of Time Spent Sitting and Walking

The KNHANES was operated by a “professional investigation team” for the purpose of conducting stable investigations. The team consists of nurses, nutritionists, and health majors, who are sent to the survey site after completing two to four weeks of training and practice, following selection. A regular training program (7 times a year) and on-site quality control are used to continuously verify the investigation capability [22].

In the 8th KNHANES, examinations were conducted in mobile examination vehicles. An informed consent form was completed by the participants after they were confirmed as the subject of the investigation. The survey items related to walking time (WT) and sitting time (ST) were administered in the mobile examination center by means of interviews. The disabled individuals were selected and surveyed according to the type of their disability via proxy responses, self-writing, and interview survey methods. As a part of the WT survey, the following question was asked: “In the last week, on days you have walked for at least 10 min, how many minutes did you walk, on average, per day?” The answer should include walking to and from work, to and from school, moving, and exercising. In the WT calculation, the time spent walking for more than 10 min at a time was added up, and the time spent walking for less than 10 min was excluded. As a part of the ST survey, the following question was asked: “How many hours do you spend sitting or lying down in a day?” The answer excluded sitting at a desk or with a friend, traveling by car, bus, train, etc., reading books, writing, playing cards or games, using the Internet, watching TV, and listening to music. 

### 2.3. Daily Energy Intake Examination

The investigator conducted the dietary survey by visiting the selected survey districts. The contents of the survey were used as indicated in [22]. The survey aims to determine what type and how much food the participants consumed throughout the previous day, from the time they woke up in the morning until they went to sleep. The respondents are asked to select one of the response views and respond with a singular number. The daily energy intake (Kcal) is then calculated, which represents the total nutrients consumed by an individual during the day. The information that the participant can respond to is the type of food and the approximate weight of food intake. Since there is a limit to directly calculating the amount of energy, nutrient intake, and food intake consumed by an individual from the surveyed information, the survey data were converted through various database (DB) linkages and processed using the KDCA. 

### 2.4. BMI Assessment

The body mass index (BMI) was calculated as the ratio of weight to squared height and categorized as normal weight (18.5 ≤ BMI < 25), obesity (25 ≤ BMI < 35), and severe obesity (35 ≤ BMI) [22]. In the 8th KNHANES, the height and weight were measured by using digital stadiometers (GL-6000-20; G-Tech, Seoul, Korea) and electronic scales (Seca 274, Seca 416; Seca, GmbH & Co, Hamburg, Germany), respectively [22]. 

### 2.5. Data Analysis

It was necessary to apply sampling weights to the 8th KNHANES database in order to ensure that the Korean population was properly represented. The missing data were treated as valid values [23]. The variables such as gender and age group were calculated as frequencies and percentages, while the variables such as the average ST per day, the average WT per day for at least 10 min at a time, and the energy intake per day were calculated as means and standard errors. The mean difference between the two groups was assessed using a *t*-test, and the frequency difference was examined using a chi-square test. To identify the leading factors affecting obesity and severe obesity in Korean adults aged 19 and older, variables such as year, gender, and age group, as well as covariates such as the average ST per day, the average WT at least 10 min at a time per day, and the energy intake per day, were adjusted simultaneously. The association between obesity or severe obesity and the adjusted variables, including covariates, was examined using multiple logistic regression analysis. All the analyses were performed using SPSS version 23.0 (IBM Corp., Armonk, NY, USA). 

### 2.6. Ethical Considerations

The 8th KNHANES was approved by the Institutional Review Board (IRB) of the KDCA [21]. In KNHANES VIII-1, the IRB number was 2018-01-03-C-A, and it was 2018-01-03-2C-A in KNHANES VIII-2. The participants in this study were not required to provide any personal information. 

## 3. Results

The findings of this study are presented in Table 1, Table 2, Table 3 and Table 4. The total number of participants was 10,059, with 5465 participants in 2019 and 4594 participants in 2020.

This study found that the prevalence of obesity among Korean adults aged 19 and over significantly increased, from 28.2% in 2019 to 30.7% in 2020. Moreover, there was a significant increase in the prevalence of severe obesity, from 5.5% in 2019 to 6.9% in 2020.

As a result of simultaneously adjusting the variables of gender, age group, the average daily ST, the average daily WT, and the average daily energy intake, the odds ratio (OR) of the obesity prevalence in Korean adults aged 19 and over in 2020 was 1.72 times higher than that in 2019, compared with the normal weight group (OR = 1.722; 95% CI, 1.034–1.329; *p* < 0.05). Moreover, in comparison to the normal weight group, the prevalence of obesity after adjusting for gender and age groups was 2.62 times higher in men than in women (OR = 2.622; 95% CI, 1.985–2.577; *p* < 0.05), and 0.62 times lower in the 19–29 age group than in the 70-and-older age group (OR = 0.617; 95% CI, 0.488–0.781, *p* < 0.05). As a result of adjusting the average daily ST, the average daily WT, and the average daily energy intake as covariates, the OR obesity prevalence increased 1.02 times with a unit increase in the ST (OR = 1.023; 95% CI, 1.006–1.041; *p* < 0.05), and 1.13 times with a unit increase in the WT (OR = 1.133; 95% CI, 1.064–1.207; *p* < 0.05); however, the daily energy intake did not show an adjusted effect (OR = 1.000; 95% CI; 1.000–1.100; *p* > 0.05) in comparison to the normal weight group.

Furthermore, the OR of severe obesity prevalence in Korean adults aged 19 and older in 2020 was 1.36 times higher than that in 2019, compared with the normal weight group (OR = 1.363; 95% CI, 1.057–1.759; *p* < 0.05). Accordingly, the prevalence of severe obesity was 1.85 times higher in men than in women (OR = 1.853; 95% CI, 1.853–1.438; *p* < 0.05), 2.71 times higher in the 19–29 age group (OR = 2.712; 95% CI, 1.578–4.663; *p* < 0.05), 3.17 times higher in the 30–39 age group (OR = 3.173; 95% CI, 1.870–5.384; *p* < 0.05), 2.51 times higher in the 40–49 age group (OR = 2.514; 95% CI, 1.477–4.278; *p* < 0.05), and 1.80 times higher in the 50–59 age group (OR = 1.798, 95% CI, 1.024–3.157; *p* < 0.05) than in the referent group, the 70-and-older age group, respectively, compared with the normal weight group. There was no significant difference in the OR of severe obesity prevalence when adjusted for the covariates such as the average daily ST, the average daily WT, and the average daily energy intake, compared with the normal weight group (*p* > 0.05).

## 4. Discussion

This study found that the OR of the obese and severely obese Korean adults aged 19 or older was 1.72 times and 1.36 times higher in 2020 after the outbreak of COVID-19 than in 2019, respectively. In order to identify the main factors influencing these findings, the relevant variables reported in previous studies were adjusted. Since the recent outbreak of COVID-19, reports on weight gain have been actively conducted; however, there have been very few previous studies on the prevalence of obesity and severe obesity in the population. Therefore, the findings of this study were compared with the previous studies assessing the prevalence of obesity and severe obesity in the population before the outbreak of COVID-19, and reporting the trend of weight gain in the population after the outbreak of COVID-19. 

Among the adjusted variables, the OR of obesity and severe obesity prevalence increased by 2.72 and 1.85 times more in men than in women. These findings contradicted those of the previous studies that reported weight gain in women during the COVID-19 self-quarantine period [15,24,25], and the pre-COVID-19 studies [26,27,28]. Moreover, these results were also inconsistent with several studies that showed that there is no gender difference [20,29,30]. In contrast to our findings, a prior study reported that the prevalence of obesity in the U.S. adult population aged 20 or older was similar for men and women from 2017 to 2018 [31]. These results were inconsistent with another prior study, which reported that the prevalence of severe obesity in the U.S. adult population aged 20 or older was higher among women from 2017 to 2018 [31]. According to the preceding studies described above, it can be concluded that women are more likely than men to gain weight following the outbreak of COVID-19. Furthermore, an earlier study suggested that women played the most significant role in predicting weight gain during the COVID-19 self-quarantine period [15]. However, in this study, men emerged as a more significant predictor of obesity than women in the Korean adult population aged 19 and over during the COVID-19 pandemic. This can be attributed to the Korean cultural practices in which women overestimate their body image and excessively practice weight management more than men [32]. Accordingly, it is suggested that a multinational joint study should be conducted in order to address the issue of whether gender is a predisposing factor for obesity. In accordance with the results regarding the prevalence of obesity, the impact of gender on the OR of severe obesity prevalence in the Korean population during the COVID-19 pandemic is due to the active weight management of women, compared with Korean adult men. It is necessary to conduct intensive research on this issue in the future.

In this study, the OR of the age-adjusted severe obesity prevalence was found to have a significant association with all the age groups, except for those in the age group of 60 to 69, compared with those aged 70 or above, after the COVID-19 outbreak. In particular, among the age groups, the OR of severe obesity prevalence in the 30–39 age group was the highest, at 3.17 times, compared with the population aged 70 and older. These findings differed from those of several previous studies that reported that age had no effect on weight gain during the COVID-19 self-quarantine period [24,25]. The OR of severe obesity prevalence in this study was inconsistent with that of a previous study, which reported that from 2017 to 2018, the prevalence of severe obesity in the U.S. adult population aged 20 and older was the highest in the adult population aged 40 to 59 [31]. According to a previous meta-analysis, the impact of age on weight gain during the COVID-19 pandemic in the adult population group is unclear [5]. There is a need for more relevant data to support the role of age as a mediator between obesity and severe obesity during the COVID-19 pandemic [5]. Nevertheless, a prior study emphasized that middle age was a predictor of weight gain after the COVID-19 outbreak [15]. Regardless, our study found that age impacts the prevalence of obesity and severe obesity. Interestingly, the adjusted covariates such as the average daily ST and the average daily WT were found to have a significant impact on the OR of obesity prevalence but not on the OR of severe obesity prevalence in the Korean adult population aged 19 and over during the COVID-19 pandemic. In other words, improving physical activity had a significant effect on the obesity prevalence OR but not on the high obesity prevalence OR. Therefore, it can be concluded that the main factors affecting the prevalence of high obesity are different; thus, other factors need to be explored in addition to physical activity. 

The OR of obesity prevalence by adjusting the average daily ST and WT revealed a significant impact of these factors on the Korean adult population aged 19 and over in 2020, compared with the normal weight group, during the COVID-19 outbreak. Based on previous studies, the most remarkable change has been the increase in sitting time or sedentary behavior per day and the decrease in walking time due to self-quarantine [24,33,34]. Additionally, we observed unfavorable impacts on the OR of obesity prevalence in the Korean adult population aged 19 or over when compared with the normal weight group. However, the OR of severe obesity prevalence had no impact on the Korean adult population aged 19 and over in 2020, compared with the normal weight group. It is presumed that this is due to the fact that there were no significant differences between the ST and WT variables before and after the outbreak of COVID-19 among Korean adults aged 19 or older, that is, between 2019 and 2020. The impacts of the adjusted ST and WT covariates on the OR of obesity prevalence are similar to the results of previous studies, but there is a curious absence of these effects on the OR of severe obesity prevalence. It is necessary to conduct intensive follow-up investigations on physical activity related to work, leisure, and movement.

Several previous studies have suggested that an increase in food intake significantly contributed to weight gain during the quarantine period of COVID-19 [5,15,19,20,25,34]. A previous study reported that the overall healthy diet score of the study participants increased as a result of the COVID-19 quarantine, which made it inevitable to prepare a home-based meal rather than eating out [19]. Similarly, our findings showed that the daily energy intake in 2020 was not statistically significant but decreased compared with that in 2019 before the COVID-19 outbreak. This is believed to be due to Koreans’ preference for consuming home-based food rather than eating out after the COVID-19 outbreak. It is, therefore, believed that the adjusted daily energy intake for the OR of obesity and severe obesity in 2020 did not have an impact on the Korean adult population 19 aged or older compared with before the outbreak. Further research is needed to examine food sources, such as home meals, convenience stores, group meals, and restaurants, as well as the frequency of eating out. Furthermore, due to the COVID-19 quarantine, a follow-up investigation is required to determine whether the individuals with normal weight accelerated to obesity or severe obesity and whether the individuals with obesity accelerated to severe obesity [19].

Based on our findings, the OR of obesity and severe obesity prevalence in 2020 in the Korean adult population aged 19 or older had a statistically significant increase, compared with the normal weight group during the COVID-19 pandemic. Adjustments were made to variables such as gender, age, daily sitting time, daily walking time, and daily energy intake. In line with the rationale of a prior study, we believe that the weight stigma is associated with weight gain due to lower physical activity and unhealthy eating habits [6]. However, it is insufficient to investigate behaviors alone to predict the cause of such weight gain [1]. Therefore, we conclude that demographic characteristics such as gender and age also influence this, requiring a longitudinal follow-up incorporating various related variables. 

This study has the following strengths: First, this study selected a representative sample of the population. It is possible to predict the overall trend of weight gain, obesity, and severe obesity in population groups and their influencing factors, and these predictions will provide the data that will help prevent and treat obesity or severe obesity at the national level. Second, this study differs from previous studies by focusing on both the period before and after the COVID-19 outbreak. This enables the identification of the prevalence of weight gain, obesity, and severe obesity during the COVID-19 pandemic. Third, unlike previous studies that focused on weight gain due to COVID-19 self-quarantine, this study identified the leading factors that contribute to the OR of obesity and severe obesity prevalence during the COVID-19 pandemic. Lastly, obesity and severe obesity were not measured by the study participants but rather by an expert using appropriate tools to ensure objective data. 

Nevertheless, this study has several shortcomings. First, it targeted the Korean adult population aged 19 or older but did not take into account any existing or sub-diseases. Furthermore, it was not confirmed whether the participants were affected by the COVID-19 pandemic. Referring to a previous report on the link between obesity and the COVID-19 outbreak [35], we propose a follow-up investigation to examine the factors influencing obesity and extreme obesity considering the underlying diseases among the study participants. Second, this study was a lateral study that pooled the Korean adult population group around the COVID-19 pandemic. Individuals with normal weight, obesity, and severe obesity during the pre-COVID-19 period require a longitudinal follow-up to determine their weight changes after the COVID-19 pandemic. Third, this study adjusted for daily sitting time, daily walking time, and daily energy intake as covariates. There were no significant differences between these three variables before and after COVID-19, and in particular, the daily energy intake had no effect on the OR of obesity and severe obesity prevalence. In addition to these covariates, further research is necessary to evaluate eating habits such as the frequency of eating out, place of eating out, and the types of meals provided at home, in restaurants, at group meals, and in convenience stores.

## 5. Conclusions

In conclusion, this study confirmed that, compared with the normal weight group, obesity and severe obesity prevalence showed a significant increase during the COVID-19 pandemic in 2020, compared with 2019. In the post-COVID-19 era, there is a national consensus that obesity or severe obesity is considered a new disease due to the outbreak of the COVID-19 pandemic. Therefore, these findings will be meaningful since they provide the basic data for national measures of obesity or severe obesity among adults. 

## Figures and Tables

**Table 1:** Demographic characteristics in Korean adults aged 19 and older.

Variables	Year 2019		Year 2020		Total	
	*N*	% (SE)	*N*	% (SE)	*N*	% (SE)
Sex						
Male	2237	49.8 (0.6)	1974	49.6 (0.7)	4311	49.7 (3.0)
Female	3128	50.2 (0.6)	2620	50.4 (0.7)	5748	50.3 (3.0)
Total	5465	100.0 (0.0)	4594	100.0 (0.0)	10,059	100.0 (0.0)
Age group						
19–29	609	17.4 (0.8)	592	17.3 (0.9)	1201	17.3 (0.6)
30–39	799	17.1 (1.0)	590	16.5 (1.1)	1389	16.8 (0.7)
40–49	979	19.4 (0.9)	759	19.0 (1.0)	1738	19.2 (0.7)
50–59	1002	19.9 (0.7)	820	19.7 (0.8)	1822	19.8 (0.2)
60–69	995	12.9 (0.7)	868	13.4 (0.7)	1863	13.2 (0.5)
≥70	1081	13.3 (0.9)	965	14.0 (1.0)	2046	13.7 (0.6)
Total	5465	100.0 (0.0)	4594	100.0 (0.0)	10,059	100.0 (0.0)

Abbreviations: *N*, number; SE, standard error.

**Table 2:** Body weight division, physical activity, and nutrition characteristics in Korean adults aged 19 and older.

Variables	2019		2020		*p* Value
	Mean ± SE		Mean ± SE		
BMI (kg/m^2^)	23.90 ± 0.06		24.20 ± 0.68		0.001
Body weight division, *N*, % (SE)					
Normal (18 ≤ BMI < 25)	3597	66.2 (0.8)	2821	62.4 (0.9)	0.005
Obesity (25 ≤ BMI < 35)	1551	28.2 (0.8)	1404	30.7 (0.8)	
Severe obesity (35 ≤ BMI)	287	5.5 (0.4)	288	6.9 (0.5)	
Total	5435	100.0 (0.0)	4513	100.0 (0.0)	
Daily sitting time	8.56 ± 0.06		8.76 ± 0.06		0.119
Daily walking time	0.65 ± 0.02		0.66 ± 0.02		0.408
Daily energy intake (Kcal)	1926.65 ± 16.30		1900.33 ± 16.00		0.077

Abbreviations: BMI, body mass index; *N*, number; SE, standard error. Pregnant women were excluded from the BMI calculation.

**Table 3:** Logistic regression analysis on obesity in Korean adults aged 19 and older.

Variables	B	SE	95% CI		*t*	*p*	OR	95% CI	
			Lower	Upper				Lower	Upper
Intercept	−1.517	0.141	−1.794	−1.241	−10.788	0.000			
Year									
2020	0.310	0.130	0.055	0.565	2.392	0.017	1.172	1.034	1.329
2019	Referent group								
Sex									
Male	0.816	0.066	0.686	0.947	12.317	0.000	2.262	1.985	2.577
Female	Referent group								
Age groups									
19–29	−0.482	0.119	−0.717	−0.247	−4.041	0.000	0.617	0.488	0.781
30–39	0.031	0.122	−0.208	0.271	0.258	0.797	1.032	0.812	1.311
40–49	0.021	0.119	−0.212	0.254	0.176	0.860	1.021	0.809	1.289
50–59	0.108	0.111	−0.112	0.327	0.964	0.336	1.114	0.894	1.387
60–69	0.153	0.113	−0.069	0.376	1.354	0.177	1.166	0.933	1.457
≥70	Referent group								
Daily sitting time	0.023	0.009	0.006	0.040	2.651	0.008	1.023	1.006	1.041
Daily walking time	0.125	0.032	0.062	0.188	3.908	0.000	1.133	1.064	1.207
Daily energy intake (Kcal)	−2.953	3.766	0.000	4.456	−0.784	0.434	1.000	1.000	1.100

Abbreviations: CI, confidence interval; OR: odds ratio; SE, standard error.

**Table 4:** Logistic regression analysis on severe obesity in Korean adults aged 19 and older.

Variables	B	SE	95% CI		*t*	*p*	OR	95% CI	
			Lower	Upper				Lower	Upper
Intercept	−3.470	0.305	−4.070	−2.870	−11.380	0.000			
Year									
2020	0.159	0.064	0.033	0.285	2.488	0.013	1.363	1.057	1.759
2019	Referent group								
Sex									
Male	0.617	0.129	0.363	0.870	4.790	0.000	1.853	1.438	2.386
Female	Referent group								
Age groups									
19–29	0.998	0.275	0.456	1.540	3.623	0.000	2.712	1.578	4.663
30–39	1.155	0.269	0.626	1.683	4.297	0.000	3.173	1.870	5.384
40–49	0.922	0.270	0.390	1.453	3.412	0.001	2.514	1.477	4.278
50–59	0.587	0.286	0.024	1.150	2.050	0.041	1.798	1.024	3.157
60–69	0.408	0.279	−0.141	0.957	1.461	0.145	1.503	0.868	2.604
≥70	Referent group								
Daily sitting time	−0.004	0.018	−0.038	0.031	−0.201	0.841	0.996	0.962	1.032
Daily walking time	0.107	0.074	−0.040	0.253	1.434	0.153	1.113	0.961	1.288
Daily energy intake (Kcal)	0.000	8.310	0.000	4.887	−1.379	0.169	1.000	1.000	1.100

Abbreviations: CI, confidence interval; OR: odds ratio; SE, standard error.

## Data Availability

There are restrictions on the availability of these data. Data were obtained from the Korea Disease Control and Prevention Agency (KDCA) and are available at https://knhanes.kdca.go.kr/knhanes/sub03/sub03_02_05.do with the permission of the KDCA (accessed on 10 April 2022).
